# Does Combined Treatment with Tranexamic Acid and Vancomycin Affect Human Chondrocytes In Vitro?

**DOI:** 10.3390/ph17121576

**Published:** 2024-11-24

**Authors:** Mike Wagenbrenner, Tizian Heinz, Philip M. Anderson, Ioannis Stratos, Joerg Arnholdt, Susanne Mayer-Wagner, Konstantin Horas, Denitsa Docheva, Boris M. Holzapfel, Maximilian Rudert, Manuel Weißenberger

**Affiliations:** 1 Department of Orthopaedic Surgery and Musculoskeletal Tissue Regeneration, University of Wuerzburg, Koenig-Ludwig-Haus, Brettreichstr. 11, 97074 Wuerzburg, Germany; mike.wagenbrenner@carealytix.com (M.W.); t-heinz.klh@uni-wuerzburg.de (T.H.); p-anderson.klh@uni-wuerzburg.de (P.M.A.); , k-horas.klh@uni-wuerzburg.de (K.H.); d-docheva.klh@uni-wuerzburg.de (D.D.); m-rudert.klh@uni-wuerzburg.de (M.R.); 2 Department of Orthopaedics and Trauma Surgery, Musculoskeletal University Center Munich (MUM), University Hospital, LMU Munich, 81377 Munich, Germany; joerg.arnholdt@med.uni-muenchen.de (J.A.); susanne.mayer@med.uni-muenchen.de (S.M.-W.); boris.holzapfel@med.uni-muenchen.de (B.M.H.)

**Keywords:** tranexamic acid, vancomycin powder, chondrocytes, total joint replacement, osteoarthritis, toxicity, viability

## Abstract

**Background:** The aim of our study was to examine the combined effects of tranexamic acid (TXA) and vancomycin powder (VP) on chondrocytes in vitro. Despite the use of TXA and VP being linked to a reduced risk of extensive postoperative blood loss and periprosthetic joint infections (PJIs) in TKA, the possible cytotoxic side effects on periarticular cell types remain unclear. **Methods:** Human chondrocytes were harvested from hyaline cartilage and expanded in monolayer culture before being simultaneously exposed to different concentrations of TXA and VP for varying exposure times. Cell viability and proliferation were assessed using an ATP assay and an Annexin 5 assay, respectively, while changes in the relative expression of chondrogenic marker genes were examined using semiquantitative RT-PCR. **Results:** The simultaneous exposure of chondrocytes to TXA and VP for more than 48 h led to a reduction in both cell viability and proliferation rates. When exposing chondrocytes to the lowest examined concentrations of both TXA (10 mg/mL) and VP (3 mg/mL), the observed effects were delayed until 96 h. However, our study found no dependencies of the observed effects on the concentrations tested. Further, we found no effects on the expression of chondrogenic marker genes. **Conclusions:** Consequently, limiting the exposure time of chondrocytes to TXA and VP in an in vitro setting to 24 h may be considered safe and could help to further improve the understanding of the safe use of substances in vivo. However, further in vitro research is required to develop a comprehensive understanding of the effects of both VP and TXA on important periarticular cell types in TKA, including chondrocytes, osteocytes, and tenocytes.

## 1. Introduction

In 2017, the rate of total knee arthroplasty (TKA) in Germany was reported to be at 223 per 100,000 people [1]. However, this finding was not limited to Germany, with the lifetime likelihood of requiring TKA in European citizens being reported to be at 10%, with numbers rising further [2,3]. Accordingly, an even more significant increase in replacement procedures of up to 90% is expected by 2050 [4].

While the rise in replacement procedures is also linked to high rates of patient dissatisfaction due to pain (41%) and functional limitations (26%), surgical complications play an important role [5]. Among the most important complications following TKA are aseptic loosening, which is associated with a decline in cement quality in cemented TKA that may be addressed by admixing bone cements, periprosthetic joint infections (PJIs) or fractures, and perioperative blood loss [5,6].

Particular attention is directed towards PJIs and perioperative blood loss due to their impact on the duration of hospital stays, cost of therapy, quality of life, and overall health [7]. The incidence of both PJIs and perioperative blood loss is not only closely tied to the need for revision surgery but is also higher following revisions [8,9]. This explains the great interest in further improving existing methods and discovering new methods to prevent the incidence of these complications in TKA.

Regarding the prevention of postoperative blood loss, the topical and systemic use of tranexamic acid (TXA) has emerged as an effective intervention. While the purely topical application of TXA is often preferred in patients with thromboembolic and cardiovascular risk factors, joint systemic and topical use is commonly performed in low-risk patients without present cardiovascular risk factors [10,11]. TXA acts by occupying the lysine binding site on plasmin, thereby limiting the conversion of plasminogen to plasmin and hindering plasmin’s fibrinolytic activity [12].

Despite ongoing debates surrounding the use of vancomycin powder (VP), its topical application has been shown to reduce the risk of PJIs following primary and revision TKA [13,14]. These findings have led to bone cement in TKA being mixed with VP to help prevent or manage PJIs in TKA [14,15]. Vancomycin functions by altering the permeability and synthesis of bacterial cell walls [13].

That being said, publications linking the individual and combined topical use of VP and TXA to potential cytotoxic effects on various periarticular cell types, including chondrocytes, osteocytes, and tenocytes, have raised concerns [16,17,18]. Although various studies have been conducted to improve the understanding of possible time- and dose-dependent cytotoxic effects of the separate use of TXA and VP on chondrocytes in vitro, there remains limited research on the joint effects of these substances on important peri- and intraarticular cell types [19]. This is particularly important, since TXA and VP are often used in a combined approach during revision procedures in TKA, and cytotoxic effects therefore may impair healthy tissue such as retropatellar cartilage [19,20,21].

Therefore, the goal of this study was to compare the effects of various exposure times (2 h, 24 h, 48 h, and 96 h) and concentrations of TXA (10 mg/mL, 50 mg/mL) and VP (3 mg/mL, 12 mg/mL, 50 mg/mL) when simultaneously exposing chondrocytes to both substances in vitro. We assessed the impact on cell viability and chondrogenic marker gene expression. These findings contribute to defining safe limits for the topical use of TXA and VP and provide insight into their effects on articular and periarticular tissues.

## 2. Results

### 2.1. ATP Assays of Chondrocytes

**Figure 1:**
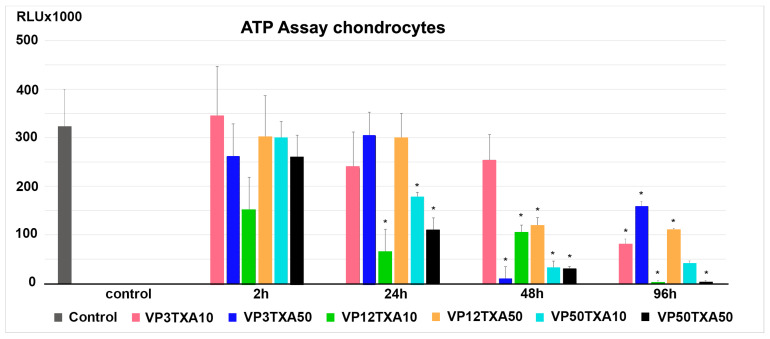
After treatment of chondrocytes with varying concentrations of TXA and VP for different exposure times, cell proliferation and viability were analyzed using ATP assays. Bar charts show mean values and standard deviations from results derived from all five donors. Statistically significant differences between varying exposure times and concentrations used were assessed using either an independent *t*-test or the Mann–Whitney U test for non-normally distributed data. * Denotes a significant difference (*p* < 0.05) compared to untreated control samples. TXA = tranexamic acid; VP = vancomycin powder; h = hours.

After being seeded in monolayer cultures and reaching confluency, the chondrocytes were exposed to different concentration combinations of TXA and VP (VP3TXA10, VP12TXA10, VP50TXA10, VP3TXA50, VP12TXA50, VP50TXA50) for 2 h, 24 h, 48 h, and 96 h. The effects of the combined treatment with TXA and VP on cell proliferation rates were assessed using ATP assays (Figure 1), while untreated chondrocytes were maintained as negative control groups (Figure 1, control).

Following 2 h of treatment with varying concentration combinations of TXA and VP, no significant effects on the proliferation rate in the chondrocyte cultures were observed in comparison to the control cultures (Figure 1, 2 h). After increasing the exposure time to 24 h, the proliferation rate was lowered significantly in the chondrocyte cultures treated with VP12TXA10, VP50TXA50, and VP50TXA10 compared to the control cultures (Figure 1, 24 h). In addition, the proliferation rate was lowered non-significantly in the chondrocyte cultures treated with VP3TXA10 for 24 h (Figure 1, 24 h). Independent of the concentration combinations of TXA and VP used, there was a trend towards a significantly lower proliferation rate in the treated chondrocyte cultures when increasing the exposure time to 48 or 96 h (Figure 1, 48 h, 96 h). However, in the cultures treated with VP3TXA10, a significant effect on proliferation was only detectable after 96 h (Figure 1, 96 h).

### 2.2. Annexin 5 Assays of Chondrocytes

**Figure 2:**
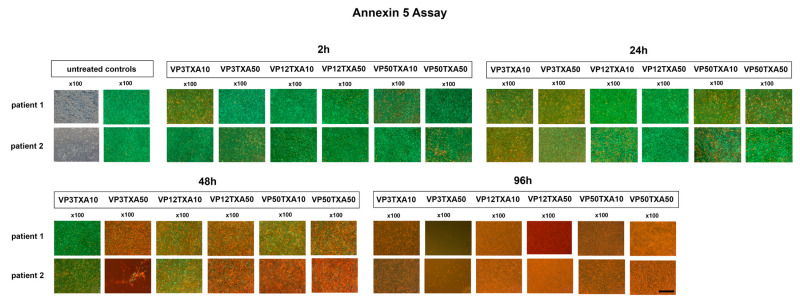
After treatment of chondrocytes with varying concentrations of TXA and VP for different exposure times, cell apoptosis was evaluated using Annexin 5 assays. After exposure to TXA and VP, chondrocyte cultures were double-stained with 6-carboxyfluorescein diacetate (CFDA) and Annexin 5. While CFDA-stained living cells appear green, Annexin 5-Cy3-stained apoptotic cells appear red. Early apoptotic cells are stained by both CFDA and Annexin 5. Within the figure, representative fluorescence microscopy images are shown (scale bar = 200 μm). CFDA = 6-carboxyfluorescein diacetate; TXA = tranexamic acid; VP = vancomycin powder; h = hours.

Cell apoptosis in the chondrocyte cultures, derived from two randomly selected donors (*n* = 2), was assessed following treatment with various concentration combinations of VP and TXA (VP3TXA10, VP12TXA10, VP50TXA10, VP3TXA50, VP12TXA50, VP50TXA50) for different exposure times (2 h, 24 h, 48 h, 96 h). Double fluorescence staining was used, with Annexin 5-Cy3 (for dead cells) and 6-carboxyfluorescein diacetate (CFDA) (for living cells) (Figure 2). Untreated cultures were used as negative controls (Figure 2, untreated controls).

The control groups as well as the chondrocyte cultures treated with different combinations of TXA and VP for 2 h and 24 h showed a distinct green fluorescence marking live cells (Figure 2, 2 h, 24 h).

Although viable cells, marked by green fluorescence, were also visible after treatment for 48 and 96 h, the assay revealed that the cultures treated with varying combinations of TXA and VP for 48 h or 96 h contained larger areas of Annexin 5-positive cells, resulting in a predominantly red fluorescence (Figure 2, 48 h, 96 h). The only exceptions were the chondrocyte cultures treated with VP3TXA10 for 48 h, which still showed a predominantly green fluorescence (Figure 2, 48 h). In addition, green fluorescence was less visible in cultures treated with TXA and VP for 96 h in comparison to cultures treated for 48 h, regardless of the concentration combination used (Figure 2, 48 h, 96 h).

### 2.3. Expression of Chondrogenic Marker Genes

**Figure 3:**
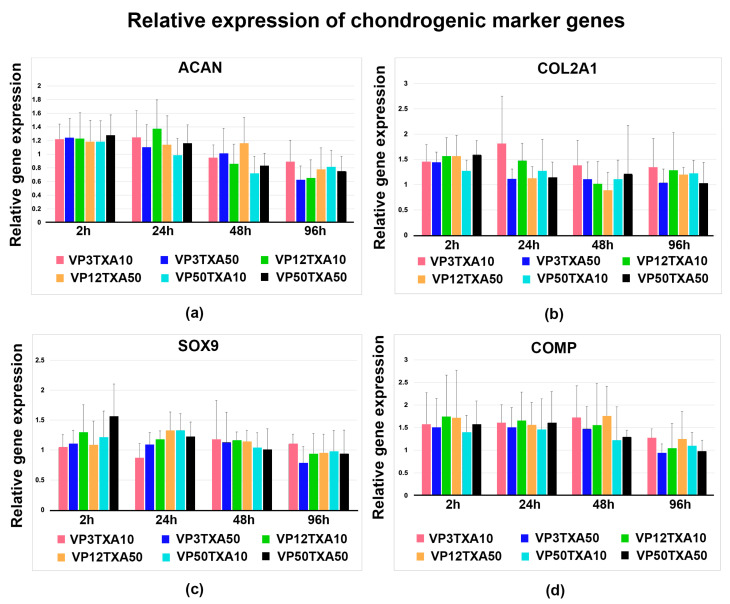
After treatment of chondrocytes with varying concentrations of TXA and VP for different exposure times, treated cultures and untreated controls isolated from five different donors (*n* = 5) were compared to examine changes in the relative expression of chondrogenic marker genes by semiquantitative RT-PCR. Bar charts represent mean values and standard deviations for the expression of chondrogenic marker genes (**a**) *ACAN*, (**b**) *COL2A1*, (**c**) *SOX9* and (**d**) *COMP*. Primer details can be found in Table 1. Statistically significant differences between the varying TXA and VP concentrations and exposure times were analyzed using an independent *t*-test or the Mann–Whitney U test in the case of non-normally distributed data. d = days; TXA = tranexamic acid; VP = vancomycin powder; h = hours.

Changes in the relative expression of chondrogenic marker genes in the chondrocyte cultures treated with TXA and VP and untreated controls were evaluated using RT-PCR (Figure 3). The chondrocytes were derived from five different donors (*n* = 5) and treated with different combinations of VP and TXA (VP3TXA10, VP12TXA10, VP50TXA10, VP3TXA50, VP12TXA50, VP50TXA50) for different exposure times (2 h, 24 h, 48 h, 96 h). Relative gene expression in all five donor samples was measured for *ACAN* (Figure 3a), *COL2A1* (Figure 3b), *SOX9* (Figure 3c), and *COMP* (Figure 3d). EEF1A1 was used as the housekeeping gene and for internal controls.

There was no significant impact of the treatment with varying concentration combinations of TXA and VP on the expression of the chondrogenic marker genes *COL2A1, ACAN, SOX9,* and *COMP* in the examined cultures, independent of the assessed exposure time (Figure 3).

However, there was a non-significant trend towards a declining relative expression of the chondrogenic marker gene *ACAN* depending on the exposure time, beginning at 48 h and continuing over 96 h, regardless of the concentrations used (Figure 3a, 48 h, 96 h). Further, the chondrocyte cultures treated with TXA and VP for 96 h also showed a non-significantly lowered relative expression of the chondrogenic marker gene *COMP* in comparison to cultures treated for shorter time periods (Figure 3d, 96 h).

## 3. Discussion

The antifibrinolytic properties of TXA have shown to significantly reduce the risk of perioperative blood loss and postoperative swelling, while VP is commonly used to prevent or treat PJIs. Consequently, both agents form the core of efforts in preventing PJIs and postoperative blood loss—two of the most common complications in primary and revision TKA [10,11,13]. Similar to hyaluronic acid, TXA may also impact infection risk by preventing the formation of local swellings and hematoma [20]. Since both substances are viewed as clinically safe and cause little to no systemic side effects during topical administration, their use during revision procedures in joint arthroplasty has gained massive popularity [14,22]. However, recent studies have now highlighted possible dose- and exposure time-dependent cytotoxic effects of both VP and TXA on important periarticular cell types, including chondrocytes, osteocytes, fibroblasts, and others [16,17,18,23]. Although some studies have considered the use of lower concentrations of TXA (up to 20 mg/mL) and VP (up to 5 mg/mL) to be safe, existing research shows great inconsistencies regarding the safe in vitro and in vivo use of both TXA and VP. Further, studies examining the combined effect of these on important articular and periarticular tissues are rare, which is the reason why our current study was conducted.

The typical effective doses for topical administration of TXA usually range between 250 mg and 3 g. Although this can lead to intraarticular concentrations of 15 mg/mL to 100 mg/mL, TXA is believed to quickly diffuse into the synovial fluid until concentrations equal plasma levels [22,24,25]. While doses for topical application of VP usually lie between 0.5 g and 4 g, the resulting intraarticular concentrations are believed to only reach around 10 mg/mL for higher doses [14,26]. In general, peak tissue concentrations may vary directly after the topical administration of both agents [14,26].

Our present in vitro study revealed a negative effect of combined treatment with both agents on proliferation rates and cell apoptosis in chondrocyte monolayer cultures when the exposure time reached at least 48 h. In contrast, there were mostly no significant effects when the treatment time was 2 h or 24 h. ATP assays highlighted a significant reduction in cell proliferation rates for all treated cultures in comparison to the untreated negative controls, beginning as early as 48 h of exposure time, when using higher concentration combinations. These findings were further underlined by Annexin 5 assays, which found an increase in apoptotic cells after treatment of the chondrocyte cultures with VP and TXA for at least 48 h. The only exceptions were the chondrocyte cultures treated with the lowest examined concentration combination of 3 mg/mL VP and 10 mg/mL TXA (VP3TXA10), which only led to a significant decline in proliferation rate and an increase in apoptotic cells after treatment for 96 h. Interestingly, topical treatment with TXA and VP did not significantly alter the relative expression of the chondrogenic marker genes *ACAN, COL2A1*, *SOX9*, and *COMP*. In summary, when jointly applying TXA and VP to chondrocyte cultures, in vitro exposure times of up to 24 h may be safe, independent of the varying concentrations used. Further decreasing concentrations for both VP and TXA to 3 mg/mL and 10 mg/mL, respectively, may allow further extension of exposure times to up to 48 h.

With the effect of the singular treatment of chondrocytes and other periarticular cell types with TXA in vitro being well studied, this is the first study looking into the combined effect of both agents on chondrocytes in vitro. Although the results of previous studies examining the cytotoxic effects of TXA on chondrocytes in vitro are mixed, most findings are in line with our current study [12,16,25,27,28,29]. Matching our current results, we found no significant effects of TXA on cell viability when limiting concentrations to 20 mg/mL or below and reducing the time of exposure to 24 h [27].

In our previous study, we found no significant effects on cell viability and chondrogenic marker gene expression when limiting TXA concentrations to 20 mg/mL and exposure time to 24 h [27]. The results published by Ambra et al. further support this hypothesis by proving that low concentrations of TXA up to 4 mg/mL had no effect on chondrocyte viability when limiting exposure time to 6 h [23]. In addition, Marmotti et al. also found no clear impact of TXA treatment on the expression of examined marker genes in chondrocytes when exposure time was limited to 48 h [29]. The same was the case for cell viability [29]. These results are also in line with in vivo studies, which found that consecutive injections of TXA 10 mg/mL in animal knees did not alter the histological features of hyaline cartilage, and the combined treatment with TXA and a-cellular scaffolds may even improve the healing time of osteochondral defects in animal knees [30,31].

On the other hand, researchers showed that increasing TXA concentrations from 20 mg/mL to 100 mg/mL may increasingly reduce cell viability in a dose- and exposure time-dependent manner and may also decrease the glycosaminoglycan content of cartilage tissue [25,28,32,33]. In addition, Parker et al. found that even short exposure to lower concentrations of 5 mg/mL to 40 mg/mL for up to 12 h may negatively affect cell viability and cellular metabolic activity in a dose-dependent manner, which does not match our current findings [17].

There is only very limited research on the effects of topical VP treatment on chondrocytes [34,35]. Contrary to our current study, researchers found that even low concentrations of vancomycin of 5 mg/mL to 12 mg/mL are toxic to human chondrocytes in vitro, even when limiting exposure time to minutes [34,35].

Despite the consistent disparities regarding the cytotoxic effects of TXA and VP on chondrocytes, which could be due to cell origin or higher passage number leading to rapid dedifferentiation of chondrocytes, most of the mentioned studies support our current results, showing that the combined treatment with TXA and VP negatively impacts chondrocyte viability and proliferation rates when increasing exposure time up to 48 h.

The limitations of our study include the small sample size. Further, the topically applied concentration of VP varies between different institutions and, at 1–2 mg/mL, may be below the minimum concentration of 3 mg/mL investigated in this study. In addition, previous research found the proliferation capacity of chondrocytes to be decreased regardless of TXA treatment when they were harvested from osteoarthritic joints [36].

According to previous research, three-dimensional cartilage models, simulating the extracellular matrix of hyaline cartilage, may also offer more resistance to topical treatment with possible cytotoxic medications [28]. Further, multiple other cells and tissues than the ones examined in our current study may affect the pharmaceutical kinetics of both TXA or VP in vivo and therefore may add further complexity to the interpretation of the findings of our current in vitro study [25].

Further research regarding more realistic cartilage models and the possible effects of TXA and VP on other functional cell types such as mesenchymal stromal cells (MSCs), osteocytes, or tenocytes is necessary to fully understand the effects of topical treatment on periarticular tissues.

## 4. Materials and Methods

### 4.1. Isolation and Culture of Chondrocytes

We obtained informed and written consent from participating patients according to the standards laid down in the Declaration of Helsinki in 1964 and as approved by the institutional review board of the University of Wuerzburg (82/08). As described in earlier studies, using a scalpel, hyaline cartilage for the isolation of chondrocytes was harvested from the femoral trochlea of five patients (*n* = 5) aged 59 to 67 (mean age: 62.3 years) within 12 h following TKA [37,38]. Cartilage was minced into small pieces of 1–2 mm^3^ and digested overnight with collagenase (0.175 U/mL; Serva Electrophoresis, Heidelberg, Germany) in Dulbecco’s Modified Eagle Medium (DMEM)/Ham’s F12 (1:1; Life Technologies GmbH, Thermo Fisher Scientific, Waltham, MA, USA). On the following day, suspended cells were centrifuged and resuspended in standard cell culture medium, before being seeded into 175 cm^2^ plastic cell culture flasks (Greiner Bio-One GmbH, Frickenhausen, Germany). 

Chondrocytes were cultured in DMEM/Ham’s F12 medium supplemented with 10% fetal bovine serum (FBS; Life Technologies GmbH) and 1% penicillin/streptomycin (PS; Life Technologies GmbH). Until reaching confluency, cell cultures were then maintained at 37 °C, 5% CO_2_, with medium changes being performed every 3 to 4 days.

### 4.2. Treatment of Human Chondrocytes with Tranexamic Acid and Vancomycin Powder

After showing confluent growth in the wells, cells were exposed to different combined concentrations of TXA (Carinopharm GmbH, Elze, Germany; 10 mg/mL, 50 mg/mL) and VP (Eberth Arzneimittel GmbH, Ursensollen, Germany; 3 mg/mL, 12 mg/mL, and 50 mg/mL) (Table 2). TXA solution was used in concentrations of topically applied TXA. As previous studies have already extensively investigated the separate effects of both drugs on chondrocytes in vitro, we decided to investigate only the combined effects of both drugs.

TXA and VP were applied after chondrocytes had reached confluence. Chondrocyte cultures were exposed to one shot of various concentrations of TXA and VP (VP3TXA10, VP12TXA10, VP50TXA10, VP3TXA50, VP12TXA50, VP50TXA50), as described above. Untreated cultures were kept as negative controls. Exposure times were 2 h, 24 h, 48 h, and 96 h.

The stock solutions of TXA (100 mg/mL) and VP (200 mg/mL) were added to standard cell culture medium. Standard cell culture medium was used as the respective negative control in untreated chondrocyte cultures. The cell cultures were treated with TXA and VP in a staggered manner according to the planned exposure time, so that the cultures could be examined together at the end. After treatment with TXA and VP, the cells were rinsed with phosphate-buffered saline (PBS) and prepared for subsequent histological, biochemical, and molecular biological analyses.

### 4.3. Biochemical Assays

After treatment of chondrocyte cultures with various concentrations of TXA and VP (VP3TXA10, VP12TXA10, VP50TXA10, VP3TXA50, VP12TXA50, VP50TXA50) for different exposure times including 2 h, 24 h, 48 h, and 96 h, we assessed cell viability using Adenosine 5′-triphosphate (ATP) assays. As described in our previous studies, we used CellTiter-Glo^®^ Luminescent Cell Viability assay (Promega GmbH, Mannheim, Germany) [27,38,39]. After exposure to TXA and VP, cells were trypsinized and seeded into 96-well plates (Greiner Bio-One GmbH) at a density of 3 × 10^3^ cells per cm² before ATP assays were performed at 2, 24, 48, and 96 h. In line with the manufacturer’s instructions, cells were mixed with 100 μL of CellTiter-Glo^®^ reagent, which consists of buffer and CellTiter-Glo^®^ substrate. After incubating cells with the reagent for 10 min, luminescence was then measured using a plate-reading luminometer (Promega GmbH).

### 4.4. Annexin 5 Assay

In line with methods described in our previous studies and the supplier’s instructions (Sigma Aldrich, St. Louis, MO, USA), we measured Annexin 5 expression as an indicator of apoptosis [39,40]. The assay employed double-labeling with the red fluorochrome Cy3.18/Annexin 5-Cy3, which binds to cells in early stages of apoptosis, and the conversion of CFDA (nonfluorescent) to 6-carboxyfluorescein (green fluorescent) by viable cells. Chondrocyte cultures were washed and fixed with 4% paraformaldehyde after being incubated in the double-labeling solution for 10 min. A fluorescence microscope (Thermo Fisher Scientific GmbH) with appropriate green and red filters was used for capturing images and qualitative assessments of both apoptotic and living cells.

### 4.5. RNA Isolation and Semiquantitative RT-PCR

Semiquantitative RT-PCR was used to examine the expression of chondrogenic marker genes collagen type II alpha 1 chain (COL2A1), aggrecan (ACAN), sex-determining region Y-box 9 (SOX9), and cartilage oligomeric matrix protein (COMP).

RNA was isolated from chondrocytes following treatment with TXA and VP using Trizol reagent (Invitrogen, Waltham, MA, USA) and additional purification steps, including DNase treatment. All procedures were carried out as outlined in the NucleoSpin^®^ RNA II kit manual (Macherey-Nagel GmbH & Co. KG, Düren, Germany). For cDNA synthesis, 1 μg of isolated RNA was combined with random hexamer primers (Thermo Fisher Scientific, Waltham, MA, USA) and Promega^®^ M-MLV reverse transcriptase (Promega GmbH). Following this, 1 μL of cDNA was used as a template in a 30 μL reaction volume, which included forward and reverse gene-specific primers (5 pmol each) and GoTaq^®^ DNA polymerase (Promega GmbH). Primer sequences, annealing temperatures, and cycle conditions for RT-PCR are provided in Table 2. As in previous studies, Elongation Factor 1α (EEF1A1) was used as the housekeeping gene [27,39,41].

Gel electrophoresis was performed on 2% agarose (Biozym Scientific GmbH, Hessisch Oldendorf, Germany) gels including 5 μL per 100 mL GelRed^®^ (Biotium, Fremont, CA, USA), using the final products of semiquantitative RT-PCR. Band densities were measured for tested genes and compared with densities for the housekeeping gene EEF1A1 to examine the relative expression of the mentioned chondrogenic marker genes.

### 4.6. Statistical Analysis

Numeric data derived from ATP assays and semiquantitative RT-PCR are presented as bar charts including mean values and standard deviations. Chondrocytes were harvested from five different patients (n = 5) with ATP assays and RT-PCR being performed in triplicate (n = 3). Normal distribution of data was assessed via Kolmogorov–Smirnov and Shapiro–Wilk tests. An independent *t*-test was employed to determine statistically significant differences between the various TXA and VP concentration combinations (VP3TXA10, VP12TXA10, VP50TXA10, VP3TXA50, VP12TXA50, VP50TXA50) and different exposure durations. Mann–Whitney U test was used for non-normally distributed data. *p*-values of less than 0.05 were considered statistically significant.

## 5. Conclusions

Our current findings reinforce the existing evidence and provide further support for the idea that limiting the exposure time to 24 h may offer a safe environment, even when both VP and TXA are used topically. Furthermore, maintaining low concentrations of TXA (10 mg/mL) and VP (3 mg/mL) may enhance safety and reduce the risk of chondrotoxicity, even with an extended exposure time of up to 48 h. However, further research is necessary to determine the possible joint effects of active ingredients on chondrocytes and other related periarticular tissues and to investigate the exact mechanisms behind the possible toxicity.

## Figures and Tables

**Table 1:** Primer sequences and product sizes for semiquantitative RT-PCR.

Gene	Primer Sequences (5′-3′)	Annealing Temperature (°C)	Product Size (Base Pairs)	Cycles	MgCl_2_
Housekeeping gene					
*EEF1A1*	Sense: AGGTGATTATCCTGAACCATCC Antisense: AAAGGTGGATAGTCTGAGAAGC	54.0	234	21	1×
Chondrogenic marker genes					
*ACAN*	Sense: GCCTTGAGCAGTTCACCTTC Antisense: CTCTTCTACGGGGACAGCAG	54.0	400	35	1×
*COL2A1*	Sense: TTTCCCAGGTCAAGATGGTC Antisense: CTTCAGCACCTGTCCACCA	51.0	155	31	1×
*SOX9*	Sense: ATCTGAAGAAGGAGAGCGAG Antisense: TCAGAAGTCTCCAGAGCTTG	60.0	263	31	1×
COMP	Sense: CAGGACGACTTTGATGCAGA Antisense: AAGCTGGAGCTGTCTGGTA	54.0	312	32	1×

**Table 2:** Concentration combinations of TXA and VP used for treatment of chondrocytes.

VP Concentration (mg/mL)	TXA Concentration (mg/mL)	Abbreviation
3	10	VP3TXA10
12	10	VP12TXA10
50	10	VP50TXA10
3	50	VP3TXA50
12	50	VP12TXA50
50	50	VP50TXA50

## Data Availability

The datasets used and analyzed during the current study are available from the first author and the corresponding author on reasonable request.

## Abbreviations

ATP, Adenosine 5′-triphosphate; CFDA, 6-carboxyfluorescein diacetate; COMP, cartilage oligomeric matrix protein; COL2A1, collagen type II alpha 1 chain; ACAN, aggrecan; d, days; DMEM, Dulbecco’s Modified Eagle Medium; EEF1A1, Elongation factor 1α; FBS, fetal bovine serum; h, hours; PS, penicillin/streptomycin; PJI, perioperative joint infection; PBS, phosphate-buffered saline; SOX9, sex-determining region Y-box 9; TKA, total knee arthroplasty; TXA, tranexamic acid; VP, vancomycin powder.

## References

[1] (2019). Health at a Glance 2019 OECD Indicators: OECD Indicators.

[2] Culliford D.J., Maskell J., Kiran A., Judge A., Javaid M.K., Cooper C., Arden N.K. (2012). The lifetime risk of total hip and knee arthroplasty: Results from the UK general practice research database. Osteoarthr. Cartil..

[3] Jonsson H., Olafsdottir S., Sigurdardottir S., Aspelund T., Eiriksdottir G., Sigurdsson S., Harris T.B., Launer L., Gudnason V. (2016). Incidence and prevalence of total joint replacements due to osteoarthritis in the elderly: Risk factors and factors associated with late life prevalence in the AGES-Reykjavik Study. BMC Musculoskelet. Disord..

[4] Klug A., Gramlich Y., Rudert M., Drees P., Hoffmann R., Weißenberger M., Kutzner K.P. (2020). The projected volume of primary and revision total knee arthroplasty will place an immense burden on future health care systems over the next 30 years. Knee Surg. Sports Traumatol. Arthrosc..

[5] Wilczyński M., Bieniek M., Krakowski P., Karpiński R. (2024). Cemented vs. Cementless Fixation in Primary Knee Replacement: A Narrative Review. Materials.

[6] Karpiński R., Szabelski J., Krakowski P., Jonak J., Falkowicz K., Jojczuk M., Nogalski A., Przekora A. (2024). Effect of various admixtures on selected mechanical properties of medium viscosity bone cements: Part 3—Glassy carbon. Compos. Struct..

[7] Postler A., Lützner C., Beyer F., Tille E., Lützner J. (2018). Analysis of Total Knee Arthroplasty revision causes. BMC Musculoskelet. Disord..

[8] Klasan A., Gerber F., Schermuksnies A., Putnis S.E., Neri T., Heyse T.J. (2021). Blood loss after revision knee arthroplasty is 1.38- to 2.17-fold higher than after primary knee arthroplasty: A retrospective analysis of 898 cases. Orthop. Traumatol. Surg. Res..

[9] Evangelopoulos D.S., Ahmad S.S., Krismer A.M., Albers C.E., Hoppe S., Kleer B., Kohl S., Ateschrang A. (2019). Periprosthetic Infection: Major Cause of Early Failure of Primary and Revision Total Knee Arthroplasty. J. Knee Surg..

[10] Zhang H., Chen J., Chen F., Que W. (2012). The effect of tranexamic acid on blood loss and use of blood products in total knee arthroplasty: A meta-analysis. Knee Surg. Sports Traumatol. Arthrosc..

[11] Sassoon A., Nam D., Jackups R., Johnson S.R., Nunley R.M., Barrack R.L. (2016). Tranexamic acid: Optimal blood loss management in surface replacement arthroplasty. Bone Jt. J..

[12] Jacob B., Kloss N., Böhle S., Kirschberg J., Zippelius T., Heinecke M., Matziolis G., Röhner E. (2020). Tranexamic acid is toxic on human chondrocytes, in vitro. J. Orthop..

[13] Liao S., Yang Z., Li X., Chen J., Liu J.G. (2022). Effects of different doses of vancomycin powder in total knee and hip arthroplasty on the periprosthetic joint infection rate: A systematic review and meta-analysis. J. Orthop. Surg. Res..

[14] Peng Z., Lin X., Kuang X., Teng Z., Lu S. (2021). The application of topical vancomycin powder for the prevention of surgical site infections in primary total hip and knee arthroplasty: A meta-analysis. Orthop. Traumatol. Surg. Res..

[15] Iarikov D., Demian H., Rubin D., Alexander J., Nambiar S. (2012). Choice and doses of antibacterial agents for cement spacers in treatment of prosthetic joint infections: Review of published studies. Clin. Infect. Dis..

[16] Bolam S.M., O’Regan-Brown A., Paul Monk A., Musson D.S., Cornish J., Munro J.T. (2020). Toxicity of tranexamic acid (TXA) to intra-articular tissue in orthopaedic surgery: A scoping review. Knee Surg. Sports Traumatol. Arthrosc..

[17] Parker J.D., Lim K.S., Kieser D.C., Woodfield T.B.F., Hooper G.J. (2018). Is tranexamic acid toxic to articular cartilage when administered topically? What is the safe dose?. Bone Jt. J..

[18] Braun J., Eckes S., Rommens P.M., Schmitz K., Nickel D., Ritz U. (2020). Toxic Effect of Vancomycin on Viability and Functionality of Different Cells Involved in Tissue Regeneration. Antibiotics.

[19] Koutalos A.A., Drakos A., Fyllos A., Doxariotis N., Varitimidis S., Malizos K.N. (2020). Does Intra-Wound Vancomycin Powder Affect the Action of Intra-Articular Tranexamic Acid in Total Joint Replacement?. Microorganisms.

[20] Benjumea A., Díaz-Navarro M., Hafian R., Cercenado E., Sánchez-Somolinos M., Vaquero J., Chana F., Muñoz P., Guembe M. (2022). Tranexamic Acid in Combination With Vancomycin or Gentamicin Has a Synergistic Effect Against Staphylococci. Front. Microbiol..

[21] Pajarinen J., Lin T.H., Nabeshima A., Jämsen E., Lu L., Nathan K., Yao Z., Goodman S.B. (2017). Mesenchymal stem cells in the aseptic loosening of total joint replacements. J. Biomed. Mater. Res. Part A.

[22] Alshryda S., Sukeik M., Sarda P., Blenkinsopp J., Haddad F.S., Mason J.M. (2014). A systematic review and meta-analysis of the topical administration of tranexamic acid in total hip and knee replacement. Bone Jt. J..

[23] Ambra L.F., de Girolamo L., Niu W., Phan A., Spector M., Gomoll A.H. (2019). No effect of topical application of tranexamic acid on articular cartilage. Knee Surg. Sports Traumatol. Arthrosc..

[24] Kim T.K., Chang C.B., Koh I.J. (2014). Practical issues for the use of tranexamic acid in total knee arthroplasty: A systematic review. Knee Surg. Sports Traumatol. Arthrosc..

[25] Goderecci R., Giusti I., Necozione S., Cinque B., D’Ascenzo S., Dolo V., Calvisi V. (2019). Short exposure to tranexamic acid does not affect, in vitro, the viability of human chondrocytes. Eur. J. Med. Res..

[26] Lawrie C.M., Kazarian G.S., Barrack T., Nunley R.M., Barrack R.L. (2021). Intra-articular administration of vancomycin and tobramycin during primary cementless total knee arthroplasty: Determination of intra-articular and serum elution profiles. Bone Jt. J..

[27] Wagenbrenner M., Heinz T., Horas K., Jakuscheit A., Arnholdt J., Mayer-Wagner S., Rudert M., Holzapfel B.M., Weißenberger M. (2020). Impact of Tranexamic Acid on Chondrocytes and Osteogenically Differentiated Human Mesenchymal Stromal Cells (hMSCs) In Vitro. J. Clin. Med..

[28] Tuttle J.R., Feltman P.R., Ritterman S.A., Ehrlich M.G. (2015). Effects of Tranexamic Acid Cytotoxicity on In Vitro Chondrocytes. Am. J. Orthop..

[29] Marmotti A., Mattia S., Mangiavini L., Bonasia D.E., Bruzzone M., Dettoni F., Rosso F., Blonna D., Rossi R., Castoldi F. (2016). Tranexamic acid effects on cartilage and synovial tissue: An in vitro study for a possible safe intra-articular use. J. Biol. Regul. Homeost. Agents.

[30] Bīrīsīk F., Bayram S., Çakmak M., Apaydın E., Erşen A. (2021). Investigation of the Effects of Intra-articular Tranexamic Acid on Intact Cartilage Tissue and Cartilage Formation in Osteochondral Defects of the Rabbit Knee: An Experimental Study. Cureus.

[31] Degirmenci E., Ozturan K.E., Sahin A.A., Yilmaz F., Kaya Y.E. (2019). Effects of tranexamic acid on the recovery of osteochondral defects treated by microfracture and acellular matrix scaffold: An experimental study. J. Orthop. Surg. Res..

[32] McLean M., McCall K., Smith I.D.M., Blyth M., Kitson S.M., Crowe L.A.N., Leach W.J., Rooney B.P., Spencer S.J., Mullen M. (2019). Tranexamic acid toxicity in human periarticular tissues. Bone Jt. Res..

[33] Pimenta F.S., de Oliveira Campos T.V., de Abreu E.S.G.M., Buzelin M.A., Nunes C.B., de Andrade M.A.P. (2023). Chondrotoxic effects of tranexamic acid and povidone-iodine on the articular cartilage of rabbits. Int. Orthop..

[34] Röhner E., Zippelius T., Böhle S., Rohe S., Matziolis G., Jacob B. (2021). Vancomycin is toxic to human chondrocytes in vitro. Arch. Orthop. Trauma Surg..

[35] Shaw K.A., Eichinger J.K., Nadig N., Parada S.A. (2018). In Vitro Effect of Vancomycin on the Viability of Articular Chondrocytes. J. Orthop. Trauma.

[36] Yang K.G., Saris D.B., Geuze R.E., van Rijen M.H., van der Helm Y.J., Verbout A.J., Creemers L.B., Dhert W.J. (2006). Altered in vitro chondrogenic properties of chondrocytes harvested from unaffected cartilage in osteoarthritic joints. Osteoarthr. Cartil..

[37] Noth U., Tuli R., Osyczka A.M., Danielson K.G., Tuan R.S. (2002). In vitro engineered cartilage constructs produced by press-coating biodegradable polymer with human mesenchymal stem cells. Tissue Eng..

[38] Steinert A.F., Proffen B., Kunz M., Hendrich C., Ghivizzani S.C., Noth U., Rethwilm A., Eulert J., Evans C.H. (2009). Hypertrophy is induced during the in vitro chondrogenic differentiation of human mesenchymal stem cells by bone morphogenetic protein-2 and bone morphogenetic protein-4 gene transfer. Arthritis Res. Ther..

[39] Steinert A.F., Weissenberger M., Kunz M., Gilbert F., Ghivizzani S.C., Gobel S., Jakob F., Noth U., Rudert M. (2012). Indian hedgehog gene transfer is a chondrogenic inducer of human mesenchymal stem cells. Arthritis Res. Ther..

[40] Weissenberger M., Weissenberger M.H., Gilbert F., Groll J., Evans C.H., Steinert A.F. (2020). Reduced hypertrophy in vitro after chondrogenic differentiation of adult human mesenchymal stem cells following adenoviral SOX9 gene delivery. BMC Musculoskelet. Disord..

[41] Steinert A.F., Kunz M., Prager P., Barthel T., Jakob F., Noth U., Murray M.M., Evans C.H., Porter R.M. (2011). Mesenchymal stem cell characteristics of human anterior cruciate ligament outgrowth cells. Tissue Eng. Part A.

